# Pilot Study on Epigenetic Response to A Mind-Body Treatment

**Published:** 2018-03-31

**Authors:** M. Cozzolino, F. Guarino, S. Castiglione, A. Cicatelli, G. Celia

**Affiliations:** 1 Department of Human, Philosophical and Educational Sciences, University of Salerno, Italy; 2 Department of Chemistry and Biology, University of Salerno, Italy; 3 International Centre of Psychology and Strategic Psychotherapy CIPPS, Salerno, Italy

**Keywords:** mind-body therapy, epigenetics, DNA methylation

## Abstract

In the last years, epigenetics and functional genomics methods to evaluate the genomic effects and mechanisms of mind-body therapies have increasingly grown.

DNA microarray technology has been used to show the involvement of the stress response pathways both in the case of disease and stress and as an effect of mind-body therapies.

In the present research, the DNA samples obtained from 20 individuals who experienced a mind-body therapeutic protocol (MBT-T), were analysed from the bio-molecular point of view by means of an epigenetic marker (MSAP molecular tool), in order to estimate the different status of methylation. The subjects were compared at 3 different times: prior to, 1 hour after, and 24 hours after the treatment.

The molecular data were processed through different biostatistics approaches: the Bayesian statistics approach, in order to estimate the clustering membership of the subjects (Structure), and the statistical estimation of the DNA methylation level (MSAP statistical tool).

The structure analysis revealed that the clusters and their membership changed among the three time points moving from higher heterogeneous distribution to higher homogeneous clusters.

Before the treatment, the subjects’ epigenetic profiles were heterogeneous; after the mind-body treatment we found that epigenetic profiles converged to homogeneous DNA methylation status.

DNA epigenetic status of the subjects was affected by the MBT-T treatment.

## I. INTRODUCTION

The field of research which studies the processes that regulate the interaction between genes and environment is called Epigenetics and it is now recognized as a new scientific approach for exploring the interaction of nature and nurture [1].

Epigenetics refers to a variety of molecular mechanisms and processes that modulate gene expression inducing long-term changes in the genome through DNA methylation, histone modification, and chromatin remodeling [2].

The substantial growth of studies in this field has increased the understanding that the environment can directly interact with the genome to influence epigenetic changes. In fact recent research show how complex epigenetic mechanisms regulate the interaction between genes and environment which in turn can modulate behavior and cognition in sickness and health [1].

The interactions gene-environment involve a special class of genes, often described as activity or experience-dependent genes, which can be activated by signals from the physical and psychosocial environment to modulate the complex functions of physiology and psychology [3, 4, 5, 6].

In the last years scientific research is studying the ways and the extent to which psychosocial stressors can have dynamic, experience-dependent epigenetic effects on gene expression [7]. At the same time an increasing number of research have investigated the effects and the mechanisms of mind-body therapies on human physiology and gene expression [8, 9, 10, 11, 12, 13, 14, 15].

This growing body of literature has mostly used functional genomics methods such as DNA microarray technology showing the involvement of the stress response pathways both in the case of disease and stress, and, in an opposite direction, as an effect of mind-body therapies.

Basically, the research documented that just as stress is able to activate specific inflammatory pathways, in the opposite direction positive psychosocial experiences, psychotherapy and mind-body treatments may improve mental and physical health through the modulation of the stress response patterns reducing the expression of pro-inflammatory genes.

Existing functional genomics studies focusing on gene expression changes related to various mind-body practices and therapies precisely showed how these kind of therapeutic approaches are able to generate an overall reduction of the expression of genes related to inflammatory response, such as NF-kB, as well as regulate numerous pathways involved in apoptosis and cell proliferation [2].

Therefore, mind-body therapeutic approaches would seem to be involved in modulating immune function and inflammatory response.

Consistent with the other studies in the field, the research we have carried out in the last ten years within the Psychosocial and Cultural Genomics approach [16] utilized DNA microarrays and bioinformatics software to explore the molecular-genomic basis of an innovative mind-body therapeutic protocol (MBT-T) with the aim to explain how psychosocial experiences can modulate gene expression to facilitate behavioral change at the molecular-genomic level.

The Psychosocial and Cultural Genomics has integrated the most recent knowledge in the field of neuroscience, psychotherapy and genomics developing both an innovative mind-body therapeutic approach, the Mind-Body Transformations Therapy (MBT-T), and an interdisciplinary research method to investigate translational mechanisms of healing on all levels from mind and brain to gene.

Specifically, the studies we carried out so far explored the hypothesis that mind-body therapies, in particular the MBT-T, could modulate experience dependent genes to reduce symptoms of the stress related disorders and facilitate mind-body healing [9, 10, 15]. Gene expression patterns characteristic of experience-dependent genes related to stem cell activation and to reduced cellular stress and inflammation were found in response to a single session of this therapeutic protocol. As observed in many other studies [8, 11], our research documented that also this kind of mind-body therapy influenced the expression of many genes mainly through a down-regulation process.

Despite an increasing number of scientific studies which support the correlation of genomic changes associated with mind-body therapeutic approaches, we have not reached yet a fully understanding of the molecular mechanisms underlying the positive effects of mind-body interventions.

Also, whereas most of the published research focus on the analysis of the genome-wide transcriptional profiling just few studies address directly the changes to the epigenome assessing for example DNA methylation changes in mind-body interventions [2].

With the aim to increase our understanding of the genomic determinants and the molecular pathways related to mind-body therapies, in this paper we present a specific study of the epigenetic response to a mind-body therapeutic approach. Specifically, we investigated the effect of the MBT-T protocol from the epigenetic point of view realizing a preliminary research in the field of mind-body treatments and epigenetic response.

## II. METHODOLOGY

### A. Introduction to materials and methods

The DNA samples, obtained from 20 individuals who experienced the mind-body therapeutic protocol (MBT-T), were analysed from the bio-molecular point of view by means of an epigenetic marker, called MSAP molecular tool, in order to estimate the different status of methylation. In particular, this analysis provided information on two different levels associated with MBT-T, *epigenetic variations* and *DNA methylation status*. The molecular data were processed through different biostatistics approaches: the *Bayesian statistics approach,* in order to estimate the clustering membership of the subjects (Structure), and the *statistical estimation of the DNA methylation level* (msap statistical tool). Both approaches were correlated to the effect of the mind-body therapeutic treatment.

The study was conducted according to the protocol and procedures here described and it was in line with the Helsinki Declaration (1996), Guidelines for Good Clinical Practice CPM/ICH/135/95-15/7/1997, compliant with Legislative Decree No.200, 6/11/2007, Implementation Directive 2005/28/EC Article 3.

The subjects taking part in the study did not run risks to their health. All the study participants completed a signed informed consent form prior to enrolment in this study.

### B. The therapeutic protocol: the Mind-Body Transformations Therapy (MBT-T)

The Mind-Body Transformations Therapy-MBT-T is an evidence-based method for the treatment of mind-body illnesses and a therapeutic approach for facilitating human resilience and resourcefulness for health and rehabilitation characterized by a structured protocol based on the four-stage creative process, the ultradian rhythms, the basic rest-activity cycle (BRAC), and the neuronal and biological plasticity [17].

MBT-T protocol can be used in a group setting or in individual sessions, it is included among mind-body therapies and uses in a new way the last epigenetic and neuroscientific findings in order to modulate the expression of genes related to an overall improvement of quality of life promoting resilience, reducing symptoms of the stress related disorders, and facilitating mind-body healing [9, 10, 15].

The mechanisms this method is based on are the use of:

- the natural Activity-Rest physiologic cycle (BRAC);
- the ultradian biological rhythms;
- the biological plasticity showed by our genes expression;
- the relaxation response (RR).

In sum the Mind-Body Transformations Therapy (MBT-T) is a therapeutic protocol which makes use of our natural biological rhythms to set the best conditions to activate inner mind-body healing processes treating the stress related dysfunctions in psychiatry, psychology and rehabilitation.

### C. Bayesian statistics (Structure): estimation of the clustering membership

The first approach here described is related to the Bayesian statistics, where the binary matrices, obtained from the presence (1) or absence (0) of the DNA fragments, were processed to estimate the structure of the population. The goal of the analysis is the comparison of the cluster membership in three different times (prior to, 1 hour after and 24 hours after the treatment) in order to assay the epigenetic variations of the subjects in response to the MBT-T treatment.

The analyses were performed independently for MspI and HpaII matrices in order to compare the effect of DNA methylation on clustering and subject membership. Structure uses a systematic Bayesian clustering approach applying Markov Chain Monte Carlo (MCMC) estimation. The MCMC process begins by randomly assigning individuals to a pre-determined number of groups, then variant frequencies are estimated in each group and individuals are re-assigned on the base of those frequency estimates. This is repeated many times, typically comprising 10,000 iterations, in the burning process that results in a progressive convergence toward reliable allele frequency estimates in each population and membership probabilities of individuals to a population.

### D. The evaluation of DNA epigenetic status at three different time points: msap statistical tool

This analysis was performed in order to estimate the DNA methylation level at three different times in relation to the MBT-T (prior to, 1 hour after and 24 hours after the treatment). The “msap” analysis allowed to evaluate, from a bioinformatics point of view, the degree of methylation of the investigated genome, by comparing the profiles obtained from EcoRI/ HpaII and EcoRI / MspI digestions. The analysis of data in msap (R) follows a strategy based on the presence or not of fragments independently for both types of loci, MSL and NML [18]. The test called “Wilcoxon rank sum test” provides statistical significance for comparing total genetic and epigenetic diversity.

## III. RESULTS

### A. Bayesian statistics (Structure): estimation of the clustering membership

First of all, we present the results of cluster membership due to epigenetic status of the subjects before the MBT-T treatment (Time A – before the treatment). Structure analyses for the MspI-A matrix revealed that the optimal K value was 3.

**Fig. 1:**
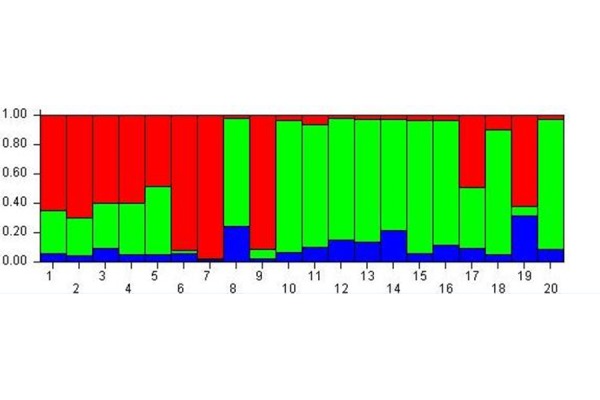
Cluster membership before MBT-T At Time A the analysis evidences 3 meta-populations from epigenetic point of view.

The analysis with K = 3 (Fig. 1) showed that all the subjects were more likely to belong to the red and green meta-populations than to the blue one. So at the beginning of experiment this analysis highlighted three meta-populations from the epigenetic point of view.

Regarding the results of the cluster membership due to the epigenetic status of the subjects 1 hour after the mind-body treatment (Time B – 1 hour after the treatment), the structure analysis of the MspI profile at Time B (MspI-B) allowed to find a single optimal value for K, which was 2.

**Fig. 2:**
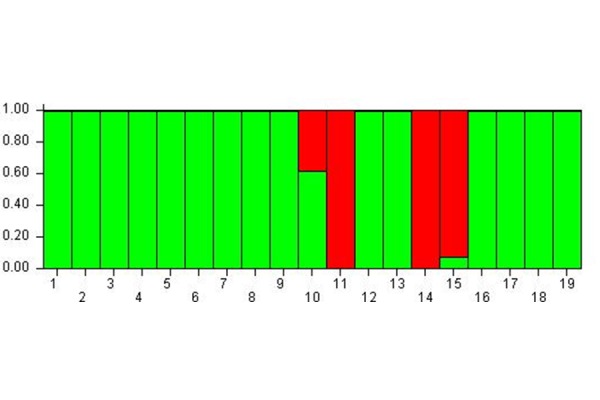
Cluster membership 1 hour after MBT-T At Time B the subjects are distributed in two well separated meta-populations.

The bar-plot shown in Fig. 2 clearly highlights that the subjects were distributed in two well separated meta-populations: most of subjects showed a high probability (100%) of belonging to the green meta-population, whereas the subject 11, 14 and 15 showed a high probability to belong to the red meta-population (100% and 90% respectively). On the contrary the subject 10 showed a good balance (60% and 40%) to belong to the green and the red meta-population.

Therefore, in time B compared to A, the subjects likely shared the same epigenetic status, in fact they almost converged to a single meta-population.

**Fig. 3:**
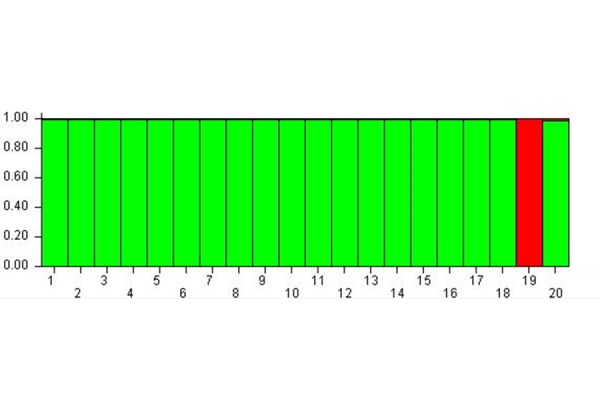
Cluster membership 24 hours after MBT-T At Time C the subjects’ epigenetic status allows to group hem within a single-meta-population except for the subject 19.

Fig. 3 shows the results of cluster membership due to the epigenetic status of the subjects 24 hours after the MBT-T treatment (Time C – 24 hours after the treatment).

The study of the MspI-C matrix allowed to find the K equal to 2. The analysis carried out considering K = 2 confirmed the trend of a shared epigenetic status for the subjects but the effect was higher than in time B. In fact, it was possible to observe the presence of two meta-populations: the green one, that included all the subjects with the exception of the subject 19 that formed the red meta-population.

For this reason, it can be stated that from time A to B, and then C, the subjects’ epigenetic status allowed to group them within a single meta-population, the green one as shown in Fig. 3, except for the subject 19 who belonged to the red meta-population. Therefore, also the subjects 10, 11, 14, 15 who previously formed the red meta-population (see Fig. 2), changed their epigenetic status and converged into the green meta-population at time C. At the same time, the subject 19, which at time B was included into the green meta-population, modified its epigenetic status passing to the red meta-population at time C.

### B. The evaluation of DNA epigenetic status at three different time points: msap statistical tool

As reported in Methodology section, the analysis of data in msap (R) followed a strategy based on the presence or not of fragments independently for both types of loci, MSL and NML and the test called “Wilcoxon rank sum test” provided statistical significance for comparing total genetic and epigenetic diversity.

The difference between MSL and NML (not methylated loci) was statistically significant at time A with a p value = 0.005, at time B and time C with p value = 0.001.

The results of the DNA msap analysis provided information about DNA methylation level. We considered the highest level of methylation for each subject. In Tab. 1 we report the number of subjects for each methylation status at three different times.

The most representative status was *double strand methylation of inner cytosine or hemi-methylation of inner cytosine* for all the time points*.* However, the results obtained from msap analysis confirmed the trend observed in the *structure analysis*.

In particular, before the MBT-T treatment (Time A) the subjects showed an heterogeneous DNA methylation level. In fact, the methylation status was distributed among the HPA-/MSP+, followed by HPA+/MSP+ and HPA-/MSP-.In the case of 1 hour after the MBT-T treatment (Time B), the DNA methylation status was balanced between HPA+/MSP+ and HPA-/MSP+. 24 hours after the treatment we found a shared DNA methylation status among the largest part of the subjects corresponding to the status HPA-/MSP+.

## IV. DISCUSSION

In this paper, we investigated the effect of a mind-body therapeutic protocol (MB-T) implemented on a group of 20 subjects from the epigenetic point of view. To reach this goal the MSAP molecular tool was employed and the data were analysed by means of diverse bio-statistical approaches.

The structure analysis revealed that the clusters and their membership changed among the three time points moving from a higher heterogeneous distribution to a higher homogeneous cluster. The results showed that 1 hour after and 24 hours after the MBT-T treatment the number of meta-populations decreased from three to two. Moreover, the membership was more homogeneous at time C than at time B. In fact, before the treatment the subjects’ epigenetic profiles were heterogeneous whereas after the mind-body treatment we found that the epigenetic profiles converged toward a homogeneous DNA methylation status. Regarding the DNA methylation level, we observed that after the treatment the *double strand methylation of inner cytosine or hemi-methylation of inner cytosine* was the most abundant DNA methylations status.

These results suggest that the DNA epigenetic status of the subjects was affected by the MBT-T treatment.

This study represents a preliminary research in the field of the relationship between mind-body therapeutic treatments and epigenetic responses. In our opinion this paper constitutes an innovative approach to genomic aspects of mind-body therapies like MBT-T. Also from a methodological point of view, we can state that the molecular markers used represent a robust, informative, cheap and reproducible methodology to perform epigenetic studies in relation to mind-body treatments.

In this study, we compared the subjects in three different times using the time A (before the MBT-T treatment) as control. In future studies it could be interesting to provide also a control group for analysing and comparing different subjects, treated or not, at the same time points. The molecular marker used does not provide information about the genes and/or pathways involved in the epigenetic response to the mind-body treatment. We foresee to employ next generation sequencing approach to overcome this limit. This will allow to identify the genes and pathways specifically involved in the epigenetic response to the MBT-T treatment.

## Figures and Tables

**Tab. 1:** DNA methylation level in the three different times (A, B, C). The table shows the number of subjects for each methylation status at three different times.

*Hpa*II	*Msp*I	Time A	Time B	Time C	Methylation status
1	1	6	8	6	No methylation
1	0	0	0	0	Hemi-methylated CHG-sites (Hemi-methylation of inner and outer cytosine)
0	1	11	8	14	Double strand methylation of inner cytosine or hemi-methylation of inner cytosine
0	0	3	3	1	Un-informative state caused either by different types of methylation or due to restriction site polymorphism

## References

[1] Hsieh J, Eisch AJ (2010). Epigenetics, Hippocampal Neurogenesis, and Neuropsychiatric Disorders: Unraveling the Genome to Understand the Mind. Neurobiol Dis.

[2] Niles H, Mehta DH, Corrigan AA, Bhasin MK, Denninger JW (2014). Functional Genomics in the Study of Mind-Body Therapies. Ochsner J.

[3] Lloyd D, Rossi EL (1992). Ultradian Rhythms in Life Processes: An Inquiry into Fundamental Principles of Chronobiology and Psychobiology.

[4] Lloyd D, Rossi EL (2008). Ultradian Rhythms From Molecules to Mind: A New Vision of Life.

[5] Ribeiro S, Goyal V, Mello C, Pavlides C (1999). Brain Gene Expression during REM Sleep Depends on prior Waking Experience. Learn Mem.

[6] Ribeiro S, Mello C, Velho T, Gardner T, Jarvis E, Pavlides C (2002). Induction of Hippocampal Long-term Potentiation During Waking Leads to Increased Extrahippocampal Zif-268 Expression During Ensuing Rapid-Eye Movement Sleep. J Neurosci.

[7] Morita K, Saito T, Ohta M, Ohmori T, Kawai K, Teshima-Kondo S, Rokutan K (2005). Expression Analysis of Psychological Stress-Associated Genes in Peripheral Blood Leukocytes. Neurosci Lett.

[8] Dusek JA, Otu HH, Wohlhueter AL, Bhasin M, Zerbini LF, Joseph MG, Benson H, Libermann TA (2008). Genomic Counter-Stress Changes Induced by the Relaxation Response. PLoS One.

[9] Rossi EL, Iannotti S, Cozzolino M, Castiglione S, Cicatelli A, Rossi KL (2008). A Pilot Study of Positive Expectations and Focused Attention via a New Protocol for Therapeutic Hypnosis Assessed with DNA Microarrays: The Creative Psychosocial Genomic Healing Experience. Sleep Hypn.

[10] Atkinson D, Iannotti S, Cozzolino M, Castiglione S, Cicatelli A, Vyas B (2010). A New Bioinformatics Paradigm for the Theory, Research, and Practice of Therapeutic Hypnosis. Am J Clin Hypn.

[11] Antoni MH, Lutgendorf SK, Blomberg B, Carver CS, Lechner S, Diaz A, Stagl J, Arevalo JM, Cole SW (2012). Cognitive-behavioral Stress Management Reverses Anxiety-related Leukocyte Transcriptional Dynamics. Biol Psychiatry.

[12] Creswell JD, Irwin MR, Burklund LJ, Lieberman MD, Arevalo JM, Ma J, Breen EC, Cole SW (2012). Mindfulness-Based Stress Reduction Training Reduces Loneliness and Proinflammatory Gene Expression in Older Adults: a Small Randomized Controlled Trial. Brain Behav Immun.

[13] Antoni MH (2013). Psychosocial Intervention Effects on Adaptation, Disease Course and Biobehavioral Processes in Cancer. Brain Behav Immun.

[14] Bhasin MK, Dusek JA, Chang BH, Joseph MG, Denninger JW, Fricchione GL, Benson H, Libermann TA Relaxation Response Induces Temporal Transcriptome Changes in Energy Metabolism, Insulin Secretion and Inflammatory Pathways. PLoS One.

[15] Cozzolino M, Cicatelli A, Fortino V, Guarino F, Tagliaferri R, Castiglione S (2015). The Mind-Body Healing Experience (MHE) is Associated with Gene Expression in Human Leukocytes. International Journal of Physical and Social Sciences.

[16] Rossi EL (2002). The Psychobiology of Gene Expression: Neuroscience and Neurogenesis in Therapeutic Hypnosis and the Healing Arts.

[17] Rossi EL (2009). The Psychosocial Genomics of Therapeutic Hypnosis, Psychotherapy, and Rehabilitation. Am J Clin Hypn.

[18] Bonin AE, Ehrich D, Manel S (2007). Statistical Analysis of Amplified Fragment Length Polymorphism Data: A Toolbox for Molecular Ecologists and Evolutionists. Mol Ecol.

